# Effects of Angiotensin-Converting Enzyme Inhibitors and Angiotensin Receptor Blockers on Angiotensin-Converting Enzyme 2 Levels: A Comprehensive Analysis Based on Animal Studies

**DOI:** 10.3389/fphar.2021.619524

**Published:** 2021-03-08

**Authors:** Gábor Kriszta, Zsófia Kriszta, Szilárd Váncsa, Péter Jenő Hegyi, Levente Frim, Bálint Erőss, Péter Hegyi, Gábor Pethő, Erika Pintér

**Affiliations:** ^1^ Department of Pharmacology and Pharmacotherapy, Medical School, University of Pécs, Pécs, Hungary; ^2^ Szentágothai Research Centre, Molecular Pharmacology Research Group, University of Pécs, Pécs, Hungary; ^3^ Department of Anaesthesiology and Intensive Therapy, Medical School, University of Pécs, Pécs, Hungary; ^4^ Institute for Translational Medicine, Medical School, University of Pécs, Pécs, Hungary; ^5^ Szentágothai Research Centre, University of Pécs, Pécs, Hungary; ^6^ Department of Pharmacology, Faculty of Pharmacy, University of Pécs, Pécs, Hungary

**Keywords:** SARS-CoV-2, angiotensin converrting enzyme, angiotensin receptor blocker, ACE2, animal study

## Abstract

Severe acute respiratory syndrome coronavirus 2 (SARS‐CoV‐2), the pathogen of coronavirus disease 2019 (COVID‐19), caused the outbreak escalated to pandemic. Reports suggested that near 1–3% of COVID‐19 cases have a fatal outcome. Angiotensin-converting enzyme inhibitors (ACEIs) and angiotensin receptor blockers (ARBs) are widely used in hypertension, heart failure and chronic kidney disease. These drugs have been reported to upregulate angiotensin converting enzyme 2 (ACE2) which produces Ang (1–7), the main counter-regulatory mediator of angiotensin II. This enzyme is also known as the receptor of SARS‐CoV‐2 promoting the cellular uptake of the virus in the airways, however, ACE2 itself proved to be protective in several experimental models of lung injury. The present study aimed to systematically review the relationship between ACEI/ARB administration and ACE2 expression in experimental models. After a comprehensive search and selection, 27 animal studies investigating ACE2 expression in the context of ACEI and ARB were identified. The majority of these papers reported increased ACE2 levels in response to ACEI/ARB treatment. This result should be interpreted in the light of the dual role of ACE2 being a promoter of viral entry to cells and a protective factor against oxidative damage in the lungs.

## Introduction

Severe acute respiratory syndrome coronavirus 2 (SARS-CoV-2), the pathogen of coronavirus disease 2019 (COVID-19), was first reported to cause human infection in Wuhan, China, in December 2019 (Tan et al., 2020). Since then, the outbreak escalated to be a pandemic, causing devastation on all continents. At the time of writing this review, there are more than 40,000,000 confirmed cases and over 1,100,000 reported deaths (WHO, 2020). Reports suggested that 1–3% of COVID-19 cases have a fatal outcome which prompted physicians and healthcare professionals to seek prognostic factors. Advanced age and cardiovascular comorbidities were confirmed to be associated with a severe form of the disease (Zhang et al., 2020) and angiotensin-converting enzyme inhibitors (ACEIs), and angiotensin receptor blockers (ARBs) widely used in the treatment of cardiovascular diseases were implicated as well (Diaz, 2020). The reason for the latter is that the angiotensin-converting enzyme 2 (ACE2), known to be the receptor of both SARS-CoV-1 and SARS-CoV-2 (Li et al., 2003; Hoffmann et al., 2020; Zhou et al., 2020), might be overexpressed in patients taking ACEIs or ARBs potentially promoting the cellular uptake of the coronavirus in the airways.

According to the classical view, angiotensin II (Ang II or Ang (1–8)), produced by the angiotensin-converting enzyme (ACE), is the major element of the renin–angiotensin system (RAS) owing to its diverse effects predominantly mediated by the angiotensin type 1 (AT_1_) receptor including vasoconstriction, a detrimental remodeling as well as oxidative stress in various tissues. ACE2, a homolog of ACE, converts Ang II to Ang (1–7) which acts on the Mas receptor and has opposite effects to those of Ang II including vasodilator, antioxidant, and anti-inflammatory actions. Thus, the overall impact of the RAS is determined by the actual balance between the ACE–Ang II and ACE2–Ang (1–7) counterparts of the system (Arendse et al., 2019).

Reviewing data from clinical studies analyzing the relationship between ACEI/ARB use and outcome of COVID-19 or ACE2 expression in humans led to the conclusion that heterogeneity and quality of these clinical studies preclude writing a reliable and conclusive review on this topic; this view is supported by some recent reports (Grover and Oberoi, 2020; Liu et al., 2020). Instead, the primary aim of the present systematic review was to examine the relationship between ACEI/ARB administration and ACE2 expression based on data from animal experiments. Although some previous reviews set a goal to summarize such animal data, they did not cover this aspect in depth because clinical studies were included as well (Kreutz et al., 2020; Sriram and Insel, 2020). Therefore, our present study can be considered the first comprehensive review on this topic which attempted also to highlight the molecular mechanisms explaining ACE2 expression changes.

## Theoretical Background

**FIGURE 1:**
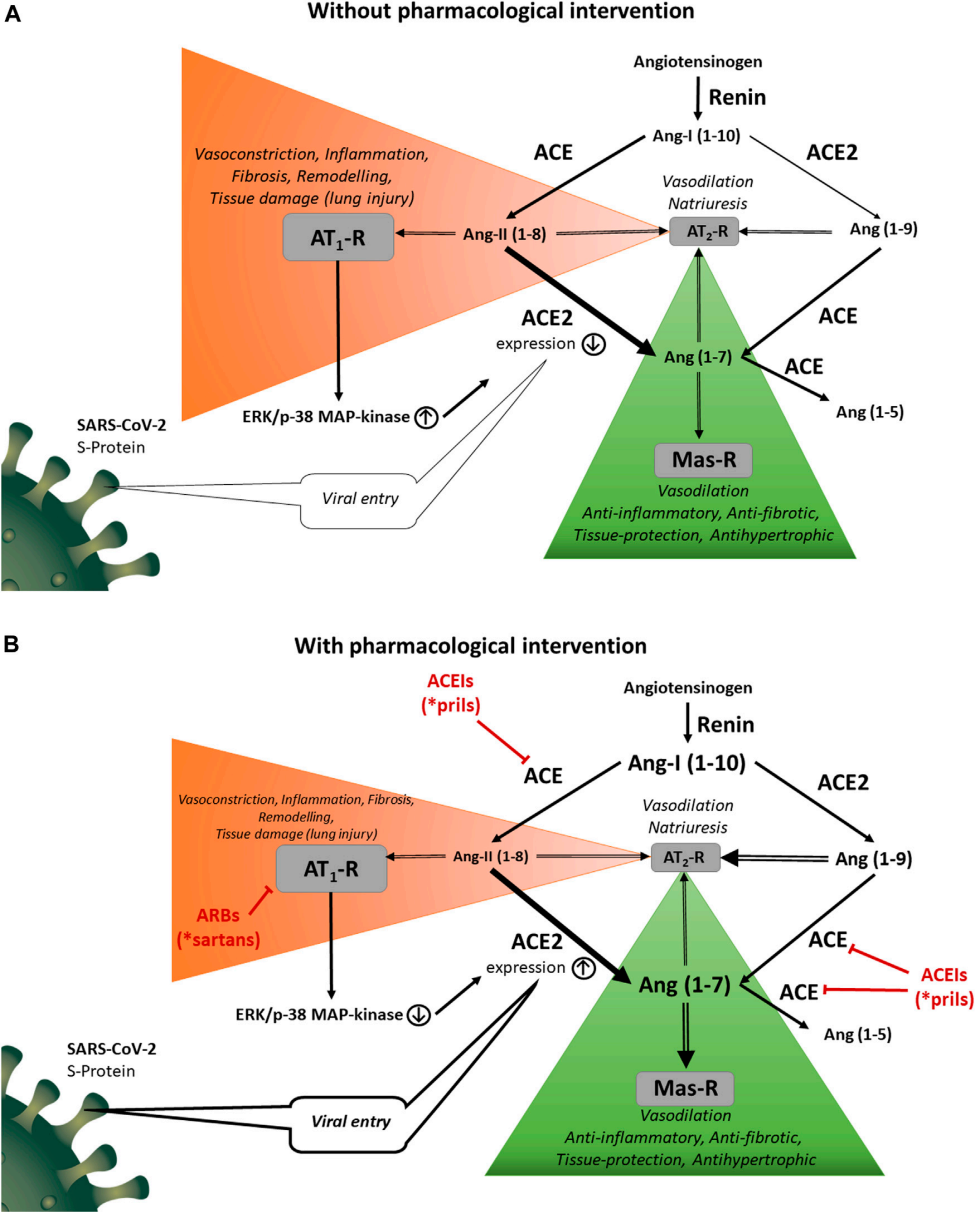
**(A)** Physiological and pathological functions of the RAS. Renin acts on angiotensinogen releasing angiotensin I (Ang I; Ang 1–10). Angiotensin converting enzyme (ACE) transforms Ang I to angiotensin II (Ang II; Ang 1 –8). Ang II activates angiotensin II type 1 (AT1) receptor causing systemic vasoconstriction, salt and water retention, hypertension, fibrosis, inflammation, remodeling and tissue damage. Ang II also acts on angiotensin II type 2 (AT2) receptor. The AT2 receptor mediates several beneficial effects; however, its expression is quite low in adults. Angiotensin converting enzyme 2 (ACE2) converts Ang I to Ang (1 –9) and Ang II to Ang (1 –7). Ang (1 –9) is converted to Ang (1 –7) and Ang (1 –7) to Ang (1 –5) by ACE. Ang (1 –7) and Ang (1 –9) are also ligands of AT2 receptors. Ang (1 –7) stimulates the Mas receptor, counteracting the Ang II‐induced AT1‐mediated harmful effects. Activation of AT1 receptors exerts signal transduction mechanisms by ERK1/2 and p38 MAPK pathways inducing downregulation of ACE2 expression. ACE2 has also non-catalytic function acting as a functional receptor for SARS‐CoV‐2. (B). Modulatory roles of ACEIs and ARBs on the RAS. ACEIs decrease the concentration of the main endogenous agonist of the AT1 receptor, Ang II; Ang (1 –8). Direct antagonism of the AT1 receptor (by ARBs) or reduction of the agonist (Ang II) concentration (by ACEIs) increases ACE2 expression with a marked reversal of ERK1/2 and p38 MAPK phosphorylation signaling pathway. Since ACE is responsible for the degradation of Ang (1 –7) to Ang (1 –5), ACEIs also increase Ang (1 –7) and Ang (1 –9) plasma levels. These peptides are agonists at AT2 and Mas receptors and play an important role in counter-regulatory effects against AT1‐mediated deleterious changes. On the other hand, ACE2 overexpression by ACEIs or ARBs potentially promotes the uptake of SARS‐CoV‐2 into the cells.

The RAS outlined in Figure 1A regulates arterial vascular responses, water and sodium homeostasis and it contributes to various pathological processes as well. Decreased renal blood flow or diminished NaCl reabsorption at the macula densa of the tubular system leads to renin release into the circulation (Davis and Freeman, 1976). Renin acts on the serum globulin angiotensinogen (AGT), cleaving the decapeptide Ang I also known as Ang (1–10) (Leonard et al., 1956). ACE, a zinc metalloprotease, which is expressed by vascular endothelial cells and epithelial cells of the kidney and the lung converts Ang I to the potent vasoconstrictor angiotensin II (Ang II; Ang (1–8)). It elicits physiological and pathophysiological actions mainly through the AT_1_ receptor which is widely expressed in the cardiovascular system. Activation of AT_1_ receptors leads to systemic vasoconstriction (fast pressor response) and aldosterone secretion with consequent salt and water retention (slow pressor response). Upon sustained overactivity of the RAS, Ang II acting on AT_1_ receptors contributes to diverse pathological processes including oxidative stress, inflammation and thrombosis (Husain et al., 2015; Düsing, 2016; Gromotowicz-Poplawska et al., 2016; Silva et al., 2017). In addition, remodeling in the organs of the cardiovascular system develops as well: in the blood vessels the amount of connective tissue is increased at the expense of contractile elements whereas the wall of the cardiac ventricles undergoes a hypertrophic transformation (Azevedo et al., 2016). Both in the vessels and the heart extracellular matrix production is increased. These alterations are partly mediated by hemodynamic effects of Ang II (vasoconstriction, increased afterload), partly by induction of specific proto-oncogenes (e.g. c-fos, c-jun) that regulate expression of various growth factors (e.g., fibroblast growth factor, platelet-derived growth factor, transforming growth factor-β). RAS is regulated by negative feedback with Ang II inhibiting the transcription as well as secretion of renin by direct action on the juxtaglomerular apparatus (Naftilan and Oparil, 1978).

Ang II can also cause vasodilatation acting on the angiotensin II type 2 (AT_2_) receptor (Ferrario, 2006). A likely mechanism of this effect is the formation of nitric oxide (NO) involving the phosphatidylinositol 3-kinase/AKT/endothelial NO synthase pathway (Santos et al., 2019). Although the AT_2_ receptor mediates numerous beneficial effects against acute and chronic cardiovascular disorders as well as fibrosis, inflammation, neurodegeneration and apoptosis (Namsolleck et al., 2014; Steckelings et al., 2017), its expression level is low in healthy adults making its role negligible, however, it is upregulated in various disease states such as atherosclerosis (Pernomian and da Silva, 2015). It is worth mentioning that under AT_1_ receptor blockade the Ang II level is increased due to lack of negative feedback on renin secretion. The elevated plasma levels of Ang II may evoke more significant effects on the AT_2_ receptor in spite of the low receptor density.

A key counter-regulatory element in the RAS system is ACE2, discovered in 2000 (Donoghue et al., 2000; Tipnis et al., 2000), which is a membrane-associated enzyme that converts Ang I to Ang 1–9 and Ang II to Ang 1–7 (Figure 1). Ang 1–9 can also be converted to Ang 1–7 by ACE or by other peptidases. Ang (1–7) stimulates the G protein-coupled receptor termed Mas which was shown to inhibit Ang II-induced cardiovascular hypertrophy and remodeling (Rice et al., 2006; Arendse et al., 2019). The downstream mechanism of the Ang (1–7)-activated Mas receptor includes the phosphatidylinositol 3-kinase/AKT pathway which induces endothelial NO synthase and the consequent increase of NO production (Sampaio et al., 2007). As mentioned above, the same mechanism of NO formation can be induced by activation of AT_2_ receptors. Ang (1–7) reduces the agonist-mediated increase in protein synthesis and mitogen-activated protein kinase (MAPK) signaling in cardiac myocytes, endothelial cells, smooth muscle cells and renal proximal tubular cells (Gallagher et al., 2008; Gopallawa and Uhal, 2016). Furthermore, G-protein-independent signaling has been revealed for the Mas receptor by interaction with postsynaptic density 95 protein (Tirupula et al., 2015). Ang (1–7) levels are increased after treatment with ARBs suggesting that Ang (1–7) may participate in the improvement of cardiac function. Furthermore, Ang (1–7) treatment blocks the Ang II-stimulated phosphorylation and activation of extracellular signal-regulated kinase 1/2 (ERK1/2) (Tallant and Clark, 2003). An alternative way for Ang (1–7) production is the cleavage of Ang I by a neutral endopeptidase called neprilysin (Yamamoto et al., 1992; Domenig et al., 2016). ACE is the primary enzyme responsible for the degradation of Ang (1–7) to Ang (1–5) (Chappell et al., 1998) which further explains the increase in Ang (1–7) and Ang (1–9) plasma levels associated with ACEIs. Ang (1–5), similarly to Ang (1–7), possesses cardioprotective properties by activating the Mas receptor with consequent release of atrial natriuretic peptide (Arendse et al., 2019). A further Ang (1–7) derivative is alamandine which contains alanine instead of aspartate as the first amino acid residue at position one (Lautner et al., 2013). Alamandine acts at the Mas-related GPCR member D (MrgD receptor) producing NO through the liver kinase B1/AMP-activated protein kinase/endothelial NO synthase pathway (Lautner et al., 2013). ACE2 activators, AT_2_ receptor agonists and Mas receptor agonists all opposing the AT_1_ receptor-mediated harmful effects of RAS have been investigated in preclinical models of drug development (Tamargo et al., 2015). ACE2 is widely expressed in the heart, kidney, testis, brain, intestine, lung and endothelial cells. Like ACE, the glycosylated ectodomain of ACE2 is cleaved by the disintegrin and metalloprotease ADAM17 from the plasma membrane and released into the circulation (shedding). Typically, soluble ACE2 cannot be measured in plasma of healthy individuals, its detectable concentration in the serum indicates an increased risk of cardiovascular disease probably reflecting enhanced shedding of ACE2 induced by elevated levels of AT II (Rice et al., 2006).

After binding of SARS-CoV-1/2 to ACE2 in the plasma membrane of type II pneumocytes through its spike protein, the virus–ACE2 complex is internalized by endocytosis resulting in viral entry to the cell as well as reduction of cell surface expression of ACE2 (Kuba et al., 2005; Hoffmann et al., 2020). It must be emphasized that the transmembrane serine protease TMPRSS2 and possibly other factor(s) are also needed for the viral entry (Matsuyama et al., 2010; Hoffmann et al., 2020). Upon virus binding to ACE2, ADAM17 cleaves the catalytically active ectodomain of ACE2 into the extracellular space (shedding) thereby further down-regulating surface ACE2 in the infected cells (Inoue et al., 2007; Haga et al., 2008). ADAM17 inhibition reduced virus replication *in vitro* suggesting that shedding is involved in viral entry (Haga et al., 2008). The soluble ACE2 may form a complex with virus particles reducing infectivity (Li et al., 2003).

## The Role of the RAS in Animal Models of Acute Lung Injury

In three experimental models (acid aspiration, sepsis induced by coecal ligation and perforation, endotoxin challenge) genetic ablation of ACE2 in mice led to an aggravation of the pathological condition (Imai et al., 2005). In the acid aspiration model it has been revealed that an overactivity of the ACE/Ang II/AT_1_ receptor pathway contributes to acute lung injury whereas activation of ACE2 can counteract it. Several subsequent studies using the lipopolysaccharide-induced acute lung injury model provided further supporting data for the protective role of ACE2 through activation of the Ang (1–7)/Mas receptor pathway (Shi et al., 2013; Li et al., 2015; Li et al., 2016; Chen et al., 2018; Liu et al., 2018; Ye and Liu, 2020). Furthermore, the beneficial role of ACE2 was also demonstrated in other forms of acute lung injury induced by bleomycin or cigarette smoke (Rey-Parra et al., 2012; Wang et al., 2015; Yu et al., 2016). ACE2 is the receptor for SARS-CoV-1 in the lung allowing virus entry to cells and it is essential for virus replication (Li et al., 2003; Kuba et al., 2005). SARS-CoV-1 infection or administration of its spike protein resulted in reduced expression of ACE2 in the lung along with worsening of lung function in both normal and lung-injured mice. It increases Ang II levels that acts through AT_1_ receptors causing/aggravating lung injury. An overactivity of the ACE/Ang II/AT_1_ pathway relative to ACE2 was shown to contribute to acute lung injury in mice in other viral infections (H5N1, H7N9, respiratory syncytial) as well (Yang et al., 2014; Zou et al., 2014; Gu et al., 2016). All these results support the view of „good ACE2, bad ACE” hypothesis regarding acute lung injury (Nicholls and Peiris, 2005).

## Animal Studies Examining the Relationship Between ACEI/ARB Administration and ACE2 Expression

After a comprehensive search and selection, 27 animal studies investigating ACE2 expression in the context of ACEI and ARB were identified. Out of the 27 studies included, we created 10 groups based on the pathological conditions or experimental models (Table 1). (For details, see the Supplementary Material).

**TABLE 1:** Animal studies with ACEI and/or ARB intervention analyzing ACE2 expression (sorted by pathological models or conditions).

Study	Species, pathogenic model or condition	ACE2 activity and/or expression modified by model or condition without intervention	Intervention	Tissue sample (RNA and/or protein)	ACE2 activity and/or expression with intervention (vs comparator group)
Healthy					
Ferrario et al.	Healthy lewis rats (8–10 weeks, male)	—	Lisinopril and/or losartan	Left ventricle	*↑ (vs vehicle)
Cano et al.	Healthy lewis rats (60 days, male)	—	Losartan	Salivary glands (RNA and protein)	-(vs healthy control)
Hamming et al.	Healthy lewis rats (male, age not spec.)	—	Lisinopril	Kidney (RNA and protein)	-(vs healthy control)
Hypertension					
Jessup et al.	mRen2 rats (congenic hypertension) (8–10 weeks, male)	N/A	Lisinopril or losartan	Heart, kidney (RNA and protein)	*↑ (vs vehicle)
Whaley-Connell et al.	mRen2 rats (congenic hypertension) (4–5 weeks, male)	—	Valsartan	Kidney (RNA and protein)	*↑ (vs mRen2 + vehicle)
Igase et al.	Spontaneously hypertensive rats (SHR) (12 weeks, male)	N/A	Olmesartan	Aorta (RNA and protein)	*↑ (vs SHR + vehicle)
Agata et al.	Stroke prone spontaneously hypertensive rats and wistar-kyoto rats (SHR) (12 weeks, male)	*↓ (kidney)	Olmesartan	Heart, kidney (RNA and protein)	*↑ (vs SHR + vehicle)
Takeda et al.	Dahl salt-sensitive rats (DS) with high salt diet (4–5 weeks, male)	**↓	Candesartan	Heart (RNA and protein)	*↑ (vs DS + vehicle)
Zhong et al.	Spontaneously hypertensive and healthy wistar-kyoto rats (SHR) (10 weeks, male)	**↓	Telmisartan	Aorta (RNA and protein)	*↑ (vs SHR + vehicle)
Yang et al.	Spontaneously hypertensive and healthy wistar kyoto rats (SHR) (4 weeks, sex not spec.)	*↓	Enalapril	Heart (RNA and protein)	*↑ (vs SHR + vehicle, mRNA)/*↓ (vs SHR + vehicle, protein)
Myocarditis					
Sukumaran et al.	Lewis rats immunized with porcine cardiac myosin (8 weeks, male)	—	Telmisartan	Heart (protein)	*↑ (vs myocarditis + vehicle)
Sukumaran et al.		*↓	Olmesartan	Heart (RNA and protein)	*↑ (vs myocarditis + vehicle)
Sukumaran et al.		*↓	Telmisartan	Heart (RNA and protein)	*↑ (vs healthy + vehicle)
Diabetes mellitus					
Graus-Nunes et al.	C57BL/6 mice with diet-induced obesity model (3 months, male)	*↓	Losartan or telmisartan	Liver (RNA and protein)	*↑ (vs high-fat diet only)
Lo et al.	Akita mice with spontaneous type 1 diabetes or akita angiotensinogen transgenic mice (11 weeks, male)	*↓	Losartan + perindopril	Heart (RNA and protein)	*↑ (vs healthy control)
Heart failure					
Wang et al.	C57BL/6 mice with cardiac remodeling after pressure overload by transverse aortic constriction (8–10 weeks, male)	*↓	Olmesartan or candesartan or telmisartan or losartan or valsartan or irbesartan	Heart (RNA and protein)	*↓ (vs heart failure + saline (cumulated))
Zhang et al.	Sprague-dawley rats with cardiac remodeling after pressure overload by transverse aortic constriction (male, age not spec.)	*↓	Losartan or enalapril	Left ventricle (RNA and protein)	*↑ (vs heart failure + vehicle)
Smoking					
Han SX et al.	Sprague dawley rats, total body smoking model (twice a day) (male, age not spec.)	*↓	Losartan	Lung (protein)	*↑ (vs smoking + vehicle)
Subtotal nephrectomy (STN)					
Velkoska et al.	Sprague dawley rats with STN (female, age not spec.)	*↓ (kidney)/**↑ (plasma)	Ramipril	Plasma, kidney (RNA and protein)	*↑ (kidney)/**↓ (plasma)
Burchill et al.	Sprague dawley rats with STN (female, age not spec.)	*↑	Ramipril	Heart (RNA and protein)	-(vs STN + vehicle)
Burrell et al.	Sprague dawley rats with STN (age and sex not spec.)	*↓ (kidney)	Ramipril	Plasma, heart, kidney (RNA and protein)	-(vs STN + vehicle, heart)/*↑ (vs STN + vehicle, kidney)
Myocardial infarction					
Ocaranza et al.	Sprague dawley rats with ligated left coronary artery (6–7 weeks, male)	*↑	Enalapril	Plasma, left ventricle (RNA and protein)	*↑ (vs MI + vehicle)
Ishiyama et al.	Lewis rats with ligated left coronary artery (8–10 weeks, male)	N/A	Losartan or olmesartan	Heart (RNA and protein)	*↑(vs MI + vehicle)
Burchill et al.	Sprague dawley rats with ligated left coronary artery (age and sex not spec.)	*↑	Ramipril and/or valsartan	Heart, plasma (RNA and protein)	*↓ (vs MI + vehicle, plasma)
Burrell et al.	Sprague dawley rats with ligated left coronary artery (age and sex not spec.)	**↑	Ramipril	Heart (RNA and protein)	-(vs MI + vehicle)
Intestinal inflammation					
Yisireyili et al.	C57BL/6 mice with stress-induced intestinal inflammation (8 weeks, male)	*↓	Irbesartan	Colon (RNA and protein)	*↑ (vs stress + vehicle)
Human angiotensiogen-insert					
Ferrario et al.	Genetically engineered sprague dawley rats to express human seq. Of AGT gene (10 weeks, male)	N/A	Valsartan	Heart (RNA and protein)	-(vs SD-AGT vehicle control)

*p < 0.05, **p < 0.01.

Three studies assessed the influence of RAS inhibitors on ACE2 expression under physiological conditions. Following 12-days long oral administration, lisinopril or losartan elevated ACE2 mRNA level in the heart of healthy rats but their combination was ineffective (Ferrario et al., 2005). Regarding cardiac ACE2 activity, losartan or the combined treatment led to an increase but lisinopril had no effect. In accord, the cardiac Ang (1–7) level was increased by either losartan or the combination. In another study, a 3-weeks long lisinopril treatment inhibited renal ACE but not ACE2 activity and increased plasma level of Ang (1–7) (Hamming et al., 2008). In the third study, losartan failed to change ACE2 levels in rat salivary glands (Cano et al., 2019).

Seven studies assessed the effects of RAS inhibitors on ACE2 levels in animal models of hypertension. Only 4 works examined the effect of the disease itself on ACE2 expression: in 3 studies reduced cardiac or aortic ACE2 expression was revealed along with signs of cardiovascular remodeling compared to normotensive control animals (Takeda et al., 2007; Zhong and Ye, 2011; Yang et al., 2014). In the remaining one study, renal but not cardiac ACE2 expression was decreased (Agata et al., 2006). Four studies using spontaneously hypertensive rats provided consonant results that ACEI or ARB treatment reduced blood pressure, diminished cardiovascular remodeling and increased cardiac or aortic ACE2 expression either partially or above levels seen in normotensive rats (Igase et al., 2005; Agata et al., 2006; Zhong and Ye, 2011; Yang et al., 2014). Also, olmesartan elevated levels of Ang (1–7) in plasma and aorta (Igase et al., 2005). In another study, the olmesartan-induced inhibition of remodeling was reduced by an antagonist of Ang (1–7) (Agata et al., 2006). In 2 studies using a congenic model of hypertension (Ren-2), ACEI or ARB treatment increased cardiac and renal ACE2 expression (Jessup et al., 2006; Whaley-Connell et al., 2006). In Dahl salt-sensitive hypertensive rats, cardiac remodeling and reduced ACE2 expression were observed and candesartan treatment inhibited both alterations (Takeda et al., 2007).

Three studies investigated the cardioprotective effects of telmisartan and olmesartan against experimental autoimmune myocarditis induced by immunization with porcine cardiac myosin in rats (Sukumaran et al., 2011; Sukumaran et al., 2012a; Sukumaran et al., 2012b). They found significantly reduced myocardial ACE2 expression. ARB treatment effectively suppressed myocardial protein and mRNA expression of inflammatory markers [CD68, iNOS, NF-kB, interleukin-1ß, interferon-α, monocyte chemotactic protein-1]. In contrast, myocardial protein levels of ACE2 and Mas receptor were upregulated in the ARB-treated group.

In a high-fat diet-induced obesity model intrahepatic ACE2 gene expression was reduced (Graus-Nunes et al., 2019). Both losartan and telmisartan significantly enhanced ACE2 mRNA levels. Modulation of the intrahepatic RAS with a preference for the ACE2/Mas axis over the ACE/AT_1_ axis after losartan or telmisartan treatments caused beneficial hepatic and metabolic effects such as reduced hepatic triacylglycerol and improved glycemic control. Another study investigated the effects of dual RAS blockade with ACEI and ARB on ACE2 expression, hypertension and renal proximal tubular cell (RPTC) apoptosis in type 1 diabetic Akita angiotensinogen (Agt)-transgenic (Tg) mice that specifically overexpress Agt in RPTCs (Lo et al., 2012). RAS blockade with losartan or perindopril normalized renal ACE2 expression and urinary Ang (1–7) levels (both of which were decreased in untreated Akita and Akita Agt-Tg) preventing hypertension, albuminuria, tubulo-interstitial fibrosis and tubular apoptosis. RAS blockade also prevented intrarenal RAS activation, hypertension and nephropathy progression in diabetes supporting the pivotal role of intrarenal ACE2 expression.

In the pressure overload model of heart failure based on a 28-days long partial aortic constriction in the rat or mouse, cardiac remodeling was accompanied by a reduced expression of cardiac ACE2 and Mas receptor along with diminished Ang (1–7) levels in the plasma (Zhang et al., 2014; Wang et al., 2016). RAS blockade by ARBs or enalapril improved remodeling and diminished or even reversed the downregulation of the ACE2/Ang (1–7)/Mas receptor axis.

Smoking induced increased right ventricular systolic pressure, thickened wall of pulmonary arteries with apparent hypertrophy along with increased Ang II and decreased ACE2 levels in the lung of rats (Han et al., 2010). Losartan administration ameliorated these effects and partially reversed the decrease of pulmonary ACE2 expression.

Experimental acute kidney injury induced by subtotal nephrectomy in rats led to a reduction in renal ACE2 activity (Velkoska et al., 2010) but a marked increase in cardiac ACE2 activity (Burchill et al., 2008). Short-term ACE inhibition by ramipril reduced blood pressure, improved renal function, regressed left ventricular hypertrophy and normalized cardiac and renal ACE2 activity (Burchill et al., 2008). Another study investigated the effect of long-term ACE inhibition on cardiac and renal ACE2 in rats in chronic kidney disease induced by subtotal nephrectomy (Burrell et al., 2012). In these animals no change in cardiac ACE2 expression was found compared to control rats. ACE inhibition with ramipril reduced blood pressure and cardiac hypertrophy but failed to change the cardiac ACE2 expression and activity.

In most studies (3 of 4) on myocardial infarction, ligation of a coronary artery increased cardiac ACE2 expression as tested on day 7 or 28 (Burrell et al., 2005; Ocaranza et al., 2006; Burchill et al., 2012). Regarding the latter time point, RAS inhibition for 28 days evoked either no effect or a decrease of ACE2 expression. Losartan or olmesartan treatment caused cardiac ACE elevation only when the ligation itself caused no alteration. In the latter case, plasma Ang (1–7) was slightly increased by ligation and further elevated by ARBs. Regarding day 56 post ligation, a decrease in cardiac ACE2 expression was detected which was prevented by an enalapril treatment for 8 weeks (Ocaranza et al., 2006).

Stress downregulated ACE2 mRNA level in the mouse colon (Yisireyili et al., 2018). Administration of irbesartan inhibited the activation of stress-induced AT_1_ pathway, reduced intestinal reactive oxygen species accumulation, inflammation and restored ACE2 expression as well (Yisireyili et al., 2018).

Plasma and cardiac expression of ACE and ACE2 were determined in genetically engineered rats (TGR(hAGT)L1623) given vehicle or valsartan (Ferrario et al., 2019). Rats expressing the human AGT gene in their genome allowed investigation of non-renin mechanisms of excess Ang II activity since rat renin is not able to convert the human AGT protein. The antihypertensive effect of valsartan after a 14-days treatment was associated with reduced left ventricular wall thickness and augmented plasma concentrations of Ang I and Ang II. Cardiac ACE2 activity was significantly higher than ACE activity in TGR (hAGT)L1623 rats but was not altered by blockade of AT_1_ receptors.

## Discussion

Recently, ACE2 has become the focus of the cardiovascular research as a counter-regulatory component of the RAS opposing most actions of Ang II by inhibiting cardiovascular hypertrophy and remodeling (Karnik et al., 2017; Arendse et al., 2019; Santos et al., 2019). Based on animal experiments, it was proposed that ACE2 is upregulated by ACE/ARB treatment. Since ACE2 was identified as the receptor of SARS-CoV-2 (Hoffmann et al., 2020; Zhou et al., 2020), ACE2 upregulation with consequently facilitated viral uptake might aggravate lung injury and fatal outcome in the case of COVID-19. Recent clinical reports (Diaz, 2020) and reviews (Fang et al., 2020) on the COVID-19 pandemic raised such concerns without systematic analysis of results from animal and human studies leading to premature conclusions and even panic among physicians and patients taking ACEIs or ARBs. However, leading international organizations, including the WHO, realized the threat of treatment discontinuation and recommended soon in the middle of March 2020 that ongoing ACEI or ARB treatment should not be stopped. In this review, a comprehensive analysis of data concerning the effects of ACEIs/ARBs on ACE2 expression/activity in animals has been performed. The majority of the studies reported increased ACE2 levels in response to ACEI/ARB treatment.

In healthy animals, ambiguous results have been obtained concerning the elevation of ACE2 expression by RAS inhibition, only one paper supporting it (Ferrario et al., 2005; Hamming et al., 2008; Cano et al., 2019). In models of various pathological conditions (hypertension (Igase et al., 2005; Agata et al., 2006; Jessup et al., 2006; Whaley-Connell et al., 2006; Takeda et al., 2007; Zhong et al., 2011; Yang et al., 2014), myocarditis (Sukumaran et al., 2011; Sukumaran et al., 2012a), diabetes (Lo et al., 2012; Graus-Nunes et al., 2019) and smoking (Han et al., 2010), details are shown in Table 1), ACEIs and/or ARBs led to normalization of decreased ACE2 expression or elevated it above the control levels. In most studies on myocardial infarction caused by coronary artery ligation ACE2 expression was increased and RAS inhibition caused a further elevation in half of the studies (Ishiyama et al., 2004; Burrell et al., 2005; Ocaranza et al., 2006; Burchill et al., 2012). In the subtotal nephrectomy models of kidney injury, ACEI treatment elevated the reduced ACE2 expression in the kidney, but it did not affect myocardial ACE2 level (Burrell et al., 2005; Burchill et al., 2008; Velkoska et al., 2010). ARB treatment increased the diminished ACE2 expression in the stress-induced colitis in mice (Yisireyili et al., 2018). In summary, it can be concluded that two-third of the animal studies provided evidence for the upregulation of ACE2 in response to ACEI or ARB treatment.

ACEI/ARB-evoked ACE2 upregulation can be explained considering the following facts: Ang II acting on AT_1_ receptors downregulates ACE2 by several mechanisms: 1) Ang II reduces ACE2 expression by triggering ERK1/2 or p38 MAPK pathways; 2) Ang II induces cleavage of the catalytic unit of ACE2 from the cell surface (shedding) by the disintegrin and metalloprotease ADAM17; 3) Ang II induces internalization of ACE2 (Kuba et al., 2010; Deshotels et al., 2014).

Consequently, the RAS blockade by ACEI/ARB leading to diminished AT_1_ receptor stimulation results in increased tissue levels of ACE2. Since ACE2 is a functional receptor for the SARS-CoV-2, ACE2 upregulation could theoretically promote viral entry into the alveolar epithelial cells. However, convincing evidence has been provided that ACE2 activity inhibits acute lung injury (Imai et al., 2008; Gopallawa and Uhal, 2014). The molecular background of the protective role of ACE2 is presumably due to the formation of Ang (1–7) which acting on the Mas receptor opposes the various detrimental effects of Ang II mediated by the AT_1_ receptor such as oxidative stress, inflammation, tissue damage leading to severe lung injury. In accord, Ang (1–7) levels are increased after treatment with ACEIs/ARBs.

It must be emphasized that in the clinical setting, any benefit of RAS-inhibiting drugs (ACEIs, ARBs) may originate from two sources. On the one hand, the reduced level of AT_1_ receptor activation itself results in desirable effects such as vasodilation, enhanced sodium and water excretion, antiinflammatory and antioxidant effects, reduction of platelet aggregation, reversal of remodeling in the cardiovascular system etc. ACE2 upregulation is a further direct consequence of the diminished level of AT_1_ receptor stimulation leading to activation of the Ang (1–7)–Mas receptor axis. As this latter signaling pathway mediates effects that are largely opposite to those of the Ang II–AT_1_ receptor axis, similar, clinically beneficial actions may be induced. It means that even if ACE2 upregulation is revealed upon ACEI or ARB use, the therapeutic effects are not necessarily due to activation of the ACE2–Ang (1–7)–Mas receptor axis.

## Conclusion

Animal studies analyzed in the present review outlined a clear picture that ACEI/ARB treatments can cause ACE2 upregulation with consequential beneficial effects considering either cardiovascular disorders or lung injury. Nevertheless, the question whether these drugs exert favorable or harmful clinical effects regarding the outcome of COVID-19 is still unanswered. Randomized, properly designed clinical trials are needed to address this issue.

## Limitations of the Study

The present systematic review is exclusively based on results from animal experiments investigating the relation between ACEI/ARB administration and ACE2 expression. ACEI/ARB-evoked ACE2 upregulation per se is not proof that the beneficial effects of these drugs are due to the ACE2 upregulation. The review does not assess anything directly associated with COVID-19. The clinical significance of the relationship between ACEI/ARB use and ACE2 expression can only be assessed in properly designed studies involving COVID-19 patients.

## Methods

This systematic review was reported according to the Preferred Reporting Items for Systematic Reviews and Meta-Analyses 2009 (see Supplementary Figure S1) (PRISMA) Statement (Liberati et al., 2009). The protocol of this study was designed following the principles of the Cochrane Handbook for Systematic Reviews of Interventions (Higgins et al., 2019) and uploaded in advance to the Zenodo pre-print server (Kriszta et al., 2020). Our aim was to investigate the effects of ACEIs and ARBs on ACE2 activity and expression in experimental *in vitro* and *in vivo* animal models.

### Search

We searched MEDLINE, Embase, Scopus and Web of Science up to 2020/05/17, with the following search key: (“angiotensin converting enzyme inhibitor” OR “angiotensin receptor blocker” OR captopril OR enalapril OR trandolapril OR quinapril OR cilazapril OR zofenopril OR ramipril OR fosinopril OR perindopril OR losartan OR valsartan OR telmisartan OR irbesartan OR olmesartan OR candesartan) AND (“angiotensin converting enzyme 2” OR “angiotensin converting enzyme related carboxypeptidase” OR ACE2 OR ACE-2 OR “peptidyl-dipeptidase A”) No language or other filters were used in the search.

### Selection

References were managed by the EndNote X9 software (Clarivate Analytics, Philadelphia, PA, United States). Following the removal of duplicates, title and abstract screening were performed by two independent reviewers to identify potentially eligible articles. Disagreements were reviewed by third review author and resolved by consensus.

### Data Extraction

Two independent reviewers extracted relevant data. The disagreements between the independent reviewers were resolved through consensus and discussion involving the senior leaders. A standardized form (Excel datasheet) was used to extract data from the included studies. For the systematic review of experimental data, the extracted information included: study characteristics, species, sex, age, strain in case of animal models, or concentration (cell cultures or *in vitro* studies), dose regimen, route of application, duration of the treatment, outcome, Angiotensin (1–7) and Angiotensin (1–9) levels.

### Data Synthesis

Extracted data from experimental studies were synthesized exclusively narratively.

### Risk of Bias Assessment

Bias assessment was performed by two authors independently using the SYRCLE’s tool (Hooijmans et al., 2014). Disagreements were resolved by a third investigator. Results of the risk of bias assessment between studies are shown in Supplementary Table S1.

## Abbreviations

ACE, angiotensin-converting enzyme; ACE2, angiotensin-converting enzyme 2; ACEIs, angiotensin-converting enzyme inhibitors; Ang II or Ang (1–8), angiotensin II; ARBs, angiotensin receptor blockers; AT_1_, angiotensin type 1; COVID-19, coronavirus disease 2019; DS, dahl salt-sensitive rats; HT, hypertension; PCR, polymerase chain reaction; RAS, renin-angiotensin system; SARS-CoV-2, severe acute respiratory syndrome coronavirus 2; SD, sprague dawley rats; SHR, spontaneously hypertensive rats; STN, subtotal nephrectomy.

## References

[B1] Agata J. Ura N. Yoshida H. Shinshi Y. Sasaki H. Hyakkoku M. (2006). Olmesartan is an angiotensin II receptor blocker with an inhibitory effect on angiotensin-converting enzyme. Hypertens. Res. 29, 865–874. 10.1291/hypres.29.865 17345786

[B2] Arendse L. B. Danser A. H. J. Poglitsch M. Touyz R. M. Burnett J. C. Llorens-Cortes C. (2019). Novel therapeutic approaches targeting the renin-angiotensin system and associated peptides in hypertension and heart failure. Pharmacol. Rev. 71, 539–570. 10.1124/pr.118.017129 31537750 PMC6782023

[B3] Azevedo P. S. Polegato B. F. Minicucci M. F. Paiva S. A. Zornoff L. A. (2016). Cardiac remodeling: concepts, clinical impact, pathophysiological mechanisms and pharmacologic treatment. Arq. Bras. Cardiol. 106, 62–69. 10.5935/abc.20160005 26647721 PMC4728597

[B4] Burchill L. Velkoska E. Dean R. G. Lew R. A. Smith A. I. Levidiotis V. (2008). Acute kidney injury in the rat causes cardiac remodelling and increases angiotensin-converting enzyme 2 expression. Exp. Physiol. 93, 622–630. 10.1113/expphysiol.2007.040386 18223026

[B5] Burchill L. J. Velkoska E. Dean R. G. Griggs K. Patel S. K. Burrell L. M. (2012). Combination renin-angiotensin system blockade and angiotensin-converting enzyme 2 in experimental myocardial infarction: implications for future therapeutic directions. Clin. Sci. 123, 649–658. 10.1042/CS20120162 22715807

[B6] Burrell L. M. Burchill L. Dean R. G. Griggs K. Patel S. K. Velkoska E. (2012). Chronic kidney disease: cardiac and renal angiotensin-converting enzyme (ACE) 2 expression in rats after subtotal nephrectomy and the effect of ACE inhibition. Exp. Physiol. 97, 477–485. 10.1113/expphysiol.2011.063156 22198016

[B7] Burrell L. M. Risvanis J. Kubota E. Dean R. G. MacDonald P. S. Lu S. (2005). Myocardial infarction increases ACE2 expression in rat and humans. Eur. Heart J. 26, 369–375. 10.1093/eurheartj/ehi114 15671045

[B8] Cano I. P. Dionisio T. J. Cestari T. M. Calvo A. M. Colombini-Ishikiriama B. L. Faria F. A. C. (2019). Losartan and isoproterenol promote alterations in the local renin-angiotensin system of rat salivary glands. PLoS One 14, e0217030. 10.1371/journal.pone.0217030 31116771 PMC6530859

[B93] Chappell M. C. Pirro N. T. Sykes A. Ferrario C. M. (1998). Metabolism of angiotensin-(1–7) by angiotensin-converting enzyme. Hypertension 31, 362–367. 9453329 10.1161/01.hyp.31.1.362

[B9] Chen Q. F. Kuang X. D. Yuan Q. F. Hao H. Zhang T. Huang Y. H. (2018). Lipoxin A4 attenuates LPS-induced acute lung injury via activation of the ACE2-Ang-(1-7)-Mas axis. Innate Immun. 24, 285–296. 10.1177/1753425918785008 29969931 PMC6830918

[B10] Davis J. Freeman R. (1976). Mechanisms regulating renin release. Physiol. Rev. 56, 1–56. 10.1152/physrev.1976.56.1.1 1108062

[B11] Deshotels M. R. Xia H. Sriramula S. Lazartigues E. Filipeanu C. M. (2014). Angiotensin II mediates angiotensin converting enzyme type 2 internalization and degradation through an angiotensin II type I receptor-dependent mechanism. Hypertension 64, 1368–1375. 10.1161/HYPERTENSIONAHA.114.03743 25225202 PMC4231883

[B12] Diaz J. H. (2020). Hypothesis: angiotensin-converting enzyme inhibitors and angiotensin receptor blockers may increase the risk of severe COVID-19. J. Trav. Med. 27 (3), taaa041. 10.1093/jtm/taaa041 PMC7184445 32186711

[B13] Domenig O. Manzel A. Grobe N. Königshausen E. Kaltenecker C. C. Kovarik J. J. (2016). Neprilysin is a mediator of alternative renin-angiotensin-system activation in the murine and human kidney. Sci. Rep. 6, 33678. 10.1038/srep33678 27649628 PMC5030486

[B14] Donoghue M. Hsieh F. Baronas E. Godbout K. Gosselin M. Stagliano N. (2000). A novel angiotensin-converting enzyme-related carboxypeptidase (ACE2) converts angiotensin I to angiotensin 1–9. Circ. Res. 87, e1–9. 10.1161/01.res.87.5.e1 10969042

[B15] Düsing R. (2016). Pharmacological interventions into the renin-angiotensin system with ACE inhibitors and angiotensin II receptor antagonists: effects beyond blood pressure lowering. Ther. Adv. Cardiovasc. Dis. 10, 151–161. 10.1177/1753944716644130 27122491 PMC5933673

[B16] Fang L. Karakiulakis G. Roth M. (2020). Are patients with hypertension and diabetes mellitus at increased risk for COVID-19 infection? Lancet Respir. Med. 8, e21. 10.1016/S2213-2600(20)30116-8 32171062 PMC7118626

[B17] Ferrario C. M. Jessup J. Chappell M. C. Averill D. B. Brosnihan K. B. Tallant E. A. (2005). Effect of angiotensin-converting enzyme inhibition and angiotensin II receptor blockers on cardiac angiotensin-converting enzyme 2. Circulation 111, 2605–2610. 10.1161/CIRCULATIONAHA.104.510461 15897343

[B18] Ferrario C. M. (2006). Role of angiotensin II in cardiovascular disease—therapeutic implications of more than a century of research. J. Renin Angiotensin Aldosterone Syst. 7, 3–14. 10.3317/jraas.2006.003 17083068

[B19] Ferrario C. M. Voncannon J. Ahmad S. Wright K. N. Roberts D. J. Wang H. (2019). Activation of the human angiotensin-(1-12)-chymase pathway in rats with human angiotensinogen gene transcripts. Front. Cardiovasc. Med. 6, 163. 10.3389/fcvm.2019.00163 31803758 PMC6872498

[B20] Gallagher P. E. Ferrario C. M. Tallant E. A. (2008). Regulation of ACE2 in cardiac myocytes and fibroblasts. Am. J. Physiol. Heart Circ. Physiol. 295, H2373–H2379. 10.1152/ajpheart.00426.2008 18849338 PMC2614534

[B21] Gopallawa I. Uhal B. D. (2016). Angiotensin-(1–7)/mas inhibits apoptosis in alveolar epithelial cells through upregulation of MAP kinase phosphatase-2. Am. J. Physiol. Lung Cell Mol. Physiol. 310, L240–L248. 10.1152/ajplung.00187.2015 26637635 PMC4888557

[B22] Gopallawa I. Uhal B. D. (2014). Molecular and cellular mechanisms of the inhibitory effects of ACE-2/ANG1-7/Mas axis on lung injury. Curr. Top. Pharmacol. 18, 71. 26146467 PMC4487538

[B23] Graus-Nunes F. Santos F. O. Marinho T. S. Miranda C. S. Barbosa-Da-Silva S. Souza-Mello V. (2019). Beneficial effects of losartan or telmisartan on the local hepatic renin-angiotensin system to counter obesity in an experimental model. World J. Hepatol. 11, 359–369. 10.4254/wjh.v11.i4.359 31114640 PMC6504859

[B24] Gromotowicz-Poplawska A. Szoka P. Kolodziejczyk P. Kramkowski K. Wojewodzka-Zelezniakowicz M. Chabielska E. (2016). New agents modulating the renin-angiotensin-aldosterone system-Will there be a new therapeutic option? Exp. Biol. Med. 241, 1888–1899. 10.1177/1535370216660211 PMC5068455 27439538

[B25] Grover A. Oberoi M. (2020). A systematic review and meta-analysis to evaluate the clinical outcomes in COVID-19 patients on angiotensin converting enzyme inhibitors or angiotensin receptor blockers. medRxiv. 10.1093/ehjcvp/pvaa064 PMC7314072 32542337

[B26] Gu H. Xie Z. Li T. Zhang S. Lai C. Zhu P. (2016). Angiotensin-converting enzyme 2 inhibits lung injury induced by respiratory syncytial virus. Sci. Rep. 6, 19840. 10.1038/srep19840 26813885 PMC4728398

[B27] Haga S. Yamamoto N. Nakai-Murakami C. Osawa Y. Tokunaga K. Sata T. (2008). Modulation of TNF-alpha-converting enzyme by the spike protein of SARS-CoV and ACE2 induces TNF-alpha production and facilitates viral entry. Proc. Natl. Acad. Sci. USA 105, 7809–7814. 10.1073/pnas.0711241105 18490652 PMC2409424

[B28] Hamming I. Van Goor H. Turner A. Rushworth C. Michaud A. Corvol P. (2008). Differential regulation of renal angiotensin-converting enzyme (ACE) and ACE2 during ACE inhibition and dietary sodium restriction in healthy rats. Exp. Physiol. 93, 631–638. 10.1113/expphysiol.2007.041855 18192334

[B29] Han S. X. He G. M. Wang T. Chen L. Ning Y. Y. Luo F. (2010). Losartan attenuates chronic cigarette smoke exposure-induced pulmonary arterial hypertension in rats: possible involvement of angiotensin-converting enzyme-2. Toxicol. Appl. Pharmacol. 245, 100–107. 10.1016/j.taap.2010.02.009 20178811 PMC7103128

[B30] Higgins J. P. Thomas J. Chandler J. Cumpston M. Li T. Page M. J. (2019). Cochrane handbook for systematic reviews of interventions. Hoboken, NJ: John Wiley and Sons. 10.1002/14651858.ED000142 PMC10284251 31643080

[B31] Hoffmann M. Kleine-Weber H. Schroeder S. Krüger N. Herrler T. Erichsen S. (2020). SARS-CoV-2 cell entry depends on ACE2 and TMPRSS2 and is blocked by a clinically proven protease inhibitor. Cell 181 (2), 271–280.e8. 10.1016/j.cell.2020.02.052 32142651 PMC7102627

[B32] Hooijmans C. R. Rovers M. M. De Vries R. B. Leenaars M. Ritskes-Hoitinga M. Langendam M. W. (2014). SYRCLE's risk of bias tool for animal studies. BMC Med. Res. Methodol. 14, 43. 10.1186/1471-2288-14-43 24667063 PMC4230647

[B33] Husain K. Hernandez W. Ansari R. A. Ferder L. (2015). Inflammation, oxidative stress and renin angiotensin system in atherosclerosis. World J. Biol. Chem. 6, 209. 10.4331/wjbc.v6.i3.209 26322175 PMC4549761

[B34] Igase M. Strawn W. B. Gallagher P. E. Geary R. L. Ferrario C. M. (2005). Angiotensin II AT1 receptors regulate ACE2 and angiotensin-(1–7) expression in the aorta of spontaneously hypertensive rats. Am. J. Physiol. Heart Circ. Physiol. 289, H1013–H1019. 10.1152/ajpheart.00068.2005 15833808

[B35] Imai Y. Kuba K. Penninger J. M. (2008). The discovery of angiotensin-converting enzyme 2 and its role in acute lung injury in mice. Exp. Physiol. 93, 543–548. 10.1113/expphysiol.2007.040048 18448662 PMC7197898

[B36] Imai Y. Kuba K. Rao S. Huan Y. Guo F. Guan B. (2005). Angiotensin-converting enzyme 2 protects from severe acute lung failure. Nature 436, 112–116. 10.1038/nature03712 16001071 PMC7094998

[B37] Inoue Y. Tanaka N. Tanaka Y. Inoue S. Morita K. Zhuang M. (2007). Clathrin-dependent entry of severe acute respiratory syndrome coronavirus into target cells expressing ACE2 with the cytoplasmic tail deleted. J. Virol. 81, 8722–8729. 10.1128/JVI.00253-07 17522231 PMC1951348

[B38] Ishiyama Y. Gallagher P. E. Averill D. B. Tallant E. A. Brosnihan K. B. Ferrario C. M. (2004). Upregulation of angiotensin-converting enzyme 2 after myocardial infarction by blockade of angiotensin II receptors. Hypertension 43, 970–976. 10.1161/01.HYP.0000124667.34652.1a 15007027

[B39] Jessup J. A. Gallagher P. E. Averill D. B. Brosnihan K. B. Tallant E. A. Chappell M. C. (2006). Effect of angiotensin II blockade on a new congenic model of hypertension derived from transgenic Ren-2 rats. Am. J. Physiol. Heart Circ. Physiol. 291, H2166–H2172. 10.1152/ajpheart.00061.2006 16766648

[B40] Karnik S. S. Singh K. D. Tirupula K. Unal H. (2017). Significance of angiotensin 1–7 coupling with MAS1 receptor and other GPCRs to the renin‐angiotensin system: IUPHAR Review 22. Br. J. Pharmacol. 174, 737–753. 10.1111/bph.13742 28194766 PMC5387002

[B41] Kreutz R. Algharably E. a. E.-H. Azizi M. Dobrowolski P. Guzik T. Januszewicz A. (2020). Hypertension, the renin–angiotensin system, and the risk of lower respiratory tract infections and lung injury: implications for COVID-19. European society of hypertension COVID-19 task force review of evidence. Cardiovasc. Res. 116, 1688–1699. 10.1093/cvr/cvaa097 32293003 PMC7184480

[B42] Kriszta G. Kriszta Z. Erőss B. Pár G. Hegyi P. J. Váncsa S. (2020). Effect of angiotensin-converting enzyme inhibitors and angiotensin receptor blockers on the angiotensin-converting enzyme 2 levels in experimental models, and clinical outcomes of COVID-19 in humans. 10.5281/zenodo.3766469

[B43] Kuba K. Imai Y. Ohto-Nakanishi T. Penninger J. M. (2010). Trilogy of ACE2: a peptidase in the renin-angiotensin system, a SARS receptor, and a partner for amino acid transporters. Pharmacol. Ther. 128, 119–128. 10.1016/j.pharmthera.2010.06.003 20599443 PMC7112678

[B44] Kuba K. Imai Y. Rao S. Gao H. Guo F. Guan B. (2005). A crucial role of angiotensin converting enzyme 2 (ACE2) in SARS coronavirus-induced lung injury. Nat. Med. 11, 875–879. 10.1038/nm1267 16007097 PMC7095783

[B45] Lautner R. Q. Villela D. C. Fraga-Silva R. A. Silva N. Verano-Braga T. Costa-Fraga F. (2013). Discovery and characterization of alamandine: a novel component of the renin-angiotensin system. Circ. Res. 112, 1104–1111. 10.1161/CIRCRESAHA.113.301077 23446738

[B46] Leonard T. Skeggs J. R. K. Shumway N. P. (1956). The preaparation and function of hypertensin converting enzyme. J. Exp. Med. 103, 295–299. 13295487 10.1084/jem.103.3.295 PMC2136590

[B47] Li W. Moore M. J. Vasilieva N. Sui J. Wong S. K. Berne M. A. (2003). Angiotensin-converting enzyme 2 is a functional receptor for the SARS coronavirus. Nature 426, 450–454. 10.1038/nature02145 14647384 PMC7095016

[B48] Li Y. Cao Y. Zeng Z. Liang M. Xue Y. Xi C. (2015). Angiotensin-converting enzyme 2/angiotensin-(1–7)/Mas axis prevents lipopolysaccharide–induced apoptosis of pulmonary microvascular endothelial cells by inhibiting JNK/NF–κB pathways. Sci. Rep. 5, 8209. 10.1038/srep08209 25644821 PMC4314638

[B49] Li Y. Zeng Z. Cao Y. Liu Y. Ping F. Liang M. (2016). Angiotensin-converting enzyme 2 prevents lipopolysaccharide-induced rat acute lung injury via suppressing the ERK1/2 and NF-κB signaling pathways. Sci. Rep. 6, 27911. 10.1038/srep27911 27302421 PMC4908402

[B50] Liberati A. Altman D. Tetzlaff J. Mulrow C. Gøtzsche P. Ioannidis J. (2009). The PRISMA statement for reporting systematic reviews and meta-analyses of studies that evaluate health care interventions: explanation and elaboration. J. Clin. Epidemiol. 62, e1–34. 10.1016/j.jclinepi.2009.06.006 19631507

[B51] Liu J. Chen Q. Liu S. Yang X. Zhang Y. Huang F. (2018). Sini decoction alleviates *E. coli* induced acute lung injury in mice via equilibrating ACE-AngII-AT1R and ACE2-Ang-(1–7)-Mas axis. Life Sci. 208, 139–148. 10.1016/j.lfs.2018.07.013 29990483

[B52] Liu X. Long C. Xiong Q. Chen C. Ma J. Su Y. (2020). Association of angiotensin converting enzyme inhibitors and angiotensin II receptor blockers with risk of COVID‐19, inflammation level, severity, and death in patients with COVID‐19: a rapid systematic review and meta‐analysis. Clin. Cardiol. 10.1002/clc.23421 PMC7436520 32757246

[B53] Lo C. S. Liu F. Shi Y. Maachi H. Chenier I. Godin N. (2012). Dual RAS blockade normalizes angiotensin-converting enzyme-2 expression and prevents hypertension and tubular apoptosis in Akita angiotensinogen-transgenic mice. Am. J. Physiol. Ren. Physiol. 302, F840–F852. 10.1152/ajprenal.00340.2011 PMC3340935 22205225

[B54] Matsuyama S. Nagata N. Shirato K. Kawase M. Takeda M. Taguchi F. (2010). Efficient activation of the severe acute respiratory syndrome coronavirus spike protein by the transmembrane protease TMPRSS2. J. Virol. 84, 12658–12664. 10.1128/JVI.01542-10 20926566 PMC3004351

[B55] Naftilan A. J. Oparil S. (1978). Inhibition of renin release from rat kidney slices by the angiotensins. Am. J. Physiol. 235, F62–F68. 10.1152/ajprenal.1978.235.1.f62 677319

[B56] Namsolleck P. Recarti C. Foulquier S. Steckelings U. M. Unger T. (2014). AT 2 receptor and tissue injury: therapeutic implications. Curr. Hypertens. Rep. 16, 416. 10.1007/s11906-013-0416-6 24414230 PMC3906548

[B57] Nicholls J. Peiris M. (2005). Good ACE, bad ACE do battle in lung injury, SARS. Nat. Med. 11, 821–822. 10.1038/nm0805-821 16079870 PMC7095949

[B58] Ocaranza M. P. Godoy I. Jalil J. E. Varas M. Collantes P. Pinto M. (2006). Enalapril attenuates downregulation of Angiotensin-converting enzyme 2 in the late phase of ventricular dysfunction in myocardial infarcted rat. Hypertension 48, 572–578. 10.1161/01.HYP.0000237862.94083.45 16908757

[B59] Pernomian L. Da Silva C. H. (2015). Current basis for discovery and development of aryl hydrocarbon receptor antagonists for experimental and therapeutic use in atherosclerosis. Eur. J. Pharmacol. 764, 118–123. 10.1016/j.ejphar.2015.06.058 26142084

[B92] Rice G. I. Jones A. L. Grant P. J. Carter A. M. Turner A. J. Hooper N. M. (2006). Circulating activities of angiotensin-converting enzyme, its homolog, angiotensin-converting enzyme 2, and neprilysin in a family study. Hypertension 48, 914–920. 17000927 10.1161/01.HYP.0000244543.91937.79

[B60] Rey-Parra G. Vadivel A. Coltan L. Hall A. Eaton F. Schuster M. (2012). Angiotensin converting enzyme 2 abrogates bleomycin-induced lung injury. J. Mol. Med. 90, 637–647. 10.1007/s00109-012-0859-2 22246130 PMC7080102

[B61] Sampaio W. O. Henrique De Castro C. Santos R. A. Schiffrin E. L. Touyz R. M. (2007). Angiotensin-(1–7) counterregulates angiotensin II signaling in human endothelial cells. Hypertension 50, 1093–1098. 10.1161/HYPERTENSIONAHA.106.084848 17984366

[B62] Santos R. A. S. Oudit G. Y. Verano-Braga T. Canta G. Steckelings U. M. Bader M. (2019). The renin-angiotensin system: going beyond the classical paradigms. Am. J. Physiol. Heart Circ. Physiol. 316, H958–H970. 10.1152/ajpheart.00723.2018 30707614 PMC7191626

[B63] Shi Y. Zhang B. Chen X. J. Xu D. Q. Wang Y. X. Dong H. Y. (2013). Osthole protects lipopolysaccharide-induced acute lung injury in mice by preventing down-regulation of angiotensin-converting enzyme 2. Eur. J. Pharm. Sci. 48, 819–824. 10.1016/j.ejps.2012.12.031 23321685

[B64] Silva S. D. Jr. Jara Z. P. Peres R. Lima L. S. Scavone C. Montezano A. C. (2017). Temporal changes in cardiac oxidative stress, inflammation and remodeling induced by exercise in hypertension: role for local angiotensin II reduction. PloS one 12, e0189535. 10.1371/journal.pone.0189535 29232407 PMC5726656

[B65] Sriram K. Insel P. A. (2020). Risks of ACE inhibitor and ARB usage in COVID‐19: evaluating the evidence. Clin. Pharmacol. Ther. 105 (2), 236–241. 10.1002/cpt.1863 PMC7264499 32320478

[B66] Steckelings U. M. Kloet A. Sumners C. (2017). Centrally mediated cardiovascular actions of the angiotensin II type 2 receptor. Trends Endocrinol. Metab. 28, 684–693. 10.1016/j.tem.2017.06.002 28733135 PMC5563271

[B67] Sukumaran V. Veeraveedu P. T. Gurusamy N. Lakshmanan A. P. Yamaguchi K. Ma M. (2012a). Telmisartan acts through the modulation of ACE-2/ANG 1–7/mas receptor in rats with dilated cardiomyopathy induced by experimental autoimmune myocarditis. Life Sci. 90, 289–300. 10.1016/j.lfs.2011.11.018 22210452

[B68] Sukumaran V. Veeraveedu P. T. Gurusamy N. Yamaguchi K. Lakshmanan A. P. Ma M. (2011). Cardioprotective effects of telmisartan against heart failure in rats induced by experimental autoimmune myocarditis through the modulation of angiotensin-converting enzyme-2/angiotensin 1–7/mas receptor axis. Int. J. Biol. Sci. 7, 1077. 10.7150/ijbs.7.1077 21927577 PMC3174385

[B69] Sukumaran V. Veeraveedu P. T. Lakshmanan A. P. Gurusamy N. Yamaguchi K. Ma M. (2012b). Olmesartan medoxomil treatment potently improves cardiac myosin-induced dilated cardiomyopathy via the modulation of ACE-2 and ANG 1–7 mas receptor. Free Radic. Res. 46, 850–860. 10.3109/10715762.2012.684878 22497476

[B70] Takeda Y. Zhu A. Yoneda T. Usukura M. Takata H. Yamagishi M. (2007). Effects of aldosterone and angiotensin II receptor blockade on cardiac angiotensinogen and angiotensin-converting enzyme 2 expression in Dahl salt-sensitive hypertensive rats. Am. J. Hypertens. 20, 1119–1124. 10.1016/j.amjhyper.2007.05.008 17903697

[B91] Tallant E. A. Clark M. A. (2003). Molecular mechanisms of inhibition of vascular growth of angiotensin (1‐7). Hypertension 42, 574–579. 12953014 10.1161/01.HYP.0000090322.55782.30

[B90] Tamargo J. Duarte J. Ruilope L. (2015). New antihypertensive drugs under development. Curr. Med. Chem. 22, 305–342. 25386825 10.2174/0929867321666141106113018

[B71] Tan W. Zhao X. Zhao X. Ma X. Wang W. Niu P. (2020). A novel coronavirus genome identified in a cluster of pneumonia cases—wuhan, China 2019−2020. China CDC Weekly 2, 61–62. 10.46234/ccdcw2020.017 PMC8393069 34594763

[B72] Tipnis S. R. Hooper N. M. Hyde R. Karran E. Christie G. Turner A. J. (2000). A human homolog of angiotensin-converting enzyme. Cloning and functional expression as a captopril-insensitive carboxypeptidase. J. Biol. Chem. 275, 33238–33243. 10.1074/jbc.M002615200 10924499

[B73] Tirupula K. C. Zhang D. Osbourne A. Chatterjee A. Desnoyer R. Willard B. (2015). MAS C-terminal tail interacting proteins identified by mass spectrometry- based proteomic approach. PLoS One 10, e0140872. 10.1371/journal.pone.0140872 26484771 PMC4618059

[B74] Velkoska E. Dean R. G. Burchill L. Levidiotis V. Burrell L. M. (2010). Reduction in renal ACE2 expression in subtotal nephrectomy in rats is ameliorated with ACE inhibition. Clin. Sci. 118, 269–279. 10.1042/CS20090318 PMC2782317 19698082

[B75] Wang L. Wang Y. Yang T. Guo Y. Sun T. (2015). Angiotensin-converting enzyme 2 attenuates bleomycin-induced lung fibrosis in mice. Cell Physiol. Biochem. 36, 697–711. 10.1159/000430131 25998889

[B76] Wang X. Ye Y. Gong H. Wu J. Yuan J. Wang S. (2016). The effects of different angiotensin II type 1 receptor blockers on the regulation of the ACE-AngII-AT1 and ACE2-Ang(1–7)-Mas axes in pressure overload-induced cardiac remodeling in male mice. J. Mol. Cell Cardiol. 97, 180–190. 10.1016/j.yjmcc.2016.05.012 27210827

[B77] Whaley-Connell A. T. Chowdhury N. A. Hayden M. R. Stump C. S. Habibi J. Wiedmeyer C. E. (2006). Oxidative stress and glomerular filtration barrier injury: role of the renin-angiotensin system in the Ren2 transgenic rat. Am. J. Physiol. Ren. Physiol. 291, F1308–F1314. 10.1152/ajprenal.00167.2006 16788142

[B78] WHO (2020). Weekly epidemiological update [Online]. Available at: https://www.who.int/publications/m/item/weekly-update-on-covid-19---16-october-2020 (Accessed October 20, 2020).

[B79] Yamamoto K. Chappell M. C. Brosnihan K. B. Ferrario C. M. (1992). *In vivo* metabolism of angiotensin I by neutral endopeptidase (EC 3.4.24.11) in spontaneously hypertensive rats. Hypertension 19, 692–696. 10.1161/01.hyp.19.6.692 1317352

[B80] Yang P. Gu H. Zhao Z. Wang W. Cao B. Lai C. (2014). Angiotensin-converting enzyme 2 (ACE2) mediates influenza H7N9 virus-induced acute lung injury. Sci. Rep. 4, 7027. 10.1038/srep07027 25391767 PMC4229671

[B81] Ye R. Liu Z. (2020). ACE2 exhibits protective effects against LPS-induced acute lung injury in mice by inhibiting the LPS-TLR4 pathway. Exp. Mol. Pathol. 113, 104350. 10.1016/j.yexmp.2019.104350 31805278

[B82] Yisireyili M. Uchida Y. Yamamoto K. Nakayama T. Cheng X. W. Matsushita T. (2018). Angiotensin receptor blocker irbesartan reduces stress-induced intestinal inflammation via AT1a signaling and ACE2-dependent mechanism in mice. Brain Behav. Immun. 69, 167–179. 10.1016/j.bbi.2017.11.010 29155324

[B83] Yu X. Lin Q. Qin X. Ruan Z. Zhou J. Lin Z. (2016). ACE2 antagonizes VEGFa to reduce vascular permeability during acute lung injury. Cell Physiol. Biochem. 38, 1055–1062. 10.1159/000443056 26938051

[B84] Zhang P. Zhu L. Cai J. Lei F. Qin J.-J. Xie J. (2020). Association of inpatient use of angiotensin converting enzyme inhibitors and angiotensin II receptor blockers with mortality among patients with hypertension hospitalized with COVID-19. Circ. Res. 126 (12), 1671–1681. 10.1161/CIRCRESAHA.120.317134 32302265 PMC7265882

[B85] Zhang Y. Li B. Wang B. Zhang J. Wu J. Morgan T. (2014). Alteration of cardiac ACE2/Mas expression and cardiac remodelling in rats with aortic constriction. Chin. J. Physiol. 57, 335–342. 10.4077/CJP.2014.BAD268 25575522

[B86] Zhong J. C. Ye J. Y. Jin H. Y. Yu X. Yu H. M. Zhu D. L. (2011). Telmisartan attenuates aortic hypertrophy in hypertensive rats by the modulation of ACE2 and profilin-1 expression. Regul. Pept. 166, 90–97. 10.1016/j.regpep.2010.09.005 20854846

[B87] Zhong J. C. Ye J. Y. Jin H. Y. Yu X. Yu H. M. Zhu D. L. (2011). Telmisartan attenuates aortic hypertrophy in hypertensive rats by the modulation of ACE2 and profilin-1 expression, Regul. Pept. 166, 90. 10.1016/j.regpep.2010.09.005 20854846

[B88] Zhou P. Yang X. L. Wang X. G. Hu B. Zhang L. Zhang W. (2020). A pneumonia outbreak associated with a new coronavirus of probable bat origin. Nature 579, 270–273. 10.1038/s41586-020-2012-7 32015507 PMC7095418

[B89] Zou Z. Yan Y. Shu Y. Gao R. Sun Y. Li X. (2014). Angiotensin-converting enzyme 2 protects from lethal avian influenza A H5N1 infections. Nat. Commun. 5, 3594–3597. 10.1038/ncomms4594 24800825 PMC7091848

