# Supplementary material for: Effects of Angiotensin-Converting Enzyme Inhibitors and Angiotensin Receptor Blockers on Angiotensin-Converting Enzyme 2 Levels: A Comprehensive Analysis Based on Animal Studies
Source: Front Pharmacol. 2021 Mar 8;12:619524. doi: 10.3389/fphar.2021.619524 (PMC7982393; doi:10.3389/fphar.2021.619524)
Supplement: Supplementary file 1 [file datasheet1.pdf]

Supplementary Material

Effects of angiotensin-converting enzyme inhibitors and angiotensin receptor blockers on angiotensin-converting enzyme 2 levels: a comprehensive analysis based on animal studies

Gábor Kriszta[1,2], Zsófia Kriszta[1,3], Szilárd Váncsa[4,5], Péter Jenő Hegyi[4], Levente Frim[4], Bálint Erőss[4], Péter Hegyi[4,5], Gábor Pethő[1,6,7,#], Erika Pintér[1,#,*]

TABLE OF CONTENT

Supplementary Table 1. Risk of bias assessment of studies inlcuded in the animal experimental systematic review using SYRCLE'S tool

Supplementary Figure 1. PRISMA flowchart of experimental studies

References

Supplementary Table 1. Risk of bias assessment of studies inlcuded in the animal experimental systematic review using SYRCLE'S tool (4)

**Authors and titles**	**Selection bias**			**Performance bias**		**Detection bias**		**Attrition bias**	**Reporting bias**	**Other**
	**1. Sequence generation**	**2. Baseline characteristics**	**3. Allocation concealment**	**4. Random housing**	**5. Blinding**	**6. Random outcome assessment**	**7. Blinding**	**8. Incomplete outcome data**	**9. Selective outcome reporting**	**10. Other sources of bias**
Ferrario CM et al. (5)	yes	yes	yes	yes	unclear	unclear	yes	yes	yes	yes
Cano IP et al. (6)	yes	yes	yes	unclear	unclear	yes	unclear	no	yes	yes
Hamming I et al. (7)	yes	yes	yes	yes	unclear	unclear	unclear	yes	yes	yes
Jessup JA et al. (8)	yes	yes	yes	unclear	unclear	unclear	unclear	yes	yes	yes
Connell W et al. (9)	yes	yes	yes	unclear	unclear	unclear	unclear	yes	yes	yes
Igase M et al. (10)	yes	yes	yes	yes	unclear	yes	unclear	unclear	yes	yes
Agata J et al. (11)	unclear	no	no	unclear	unclear	unclear	yes	yes	yes	no
Takeda Y et al. (12)	unclear	yes	yes	yes	unclear	unclear	unclear	yes	yes	yes
Zhong JC et al. (13)	yes	no	yes	yes	unclear	unclear	unclear	unclear	yes	yes
Yang Z et al. (14)	yes	no	no	unclear	unclear	unclear	unclear	unclear	yes	yes
Sukumaran V et al. (15)	unclear	yes	yes	yes	unclear	unclear	unclear	unclear	yes	unclear
Sukumaran V et al. (16)	unclear	yes	yes	yes	unclear	unclear	unclear	yes	yes	yes
Sukumaran V et al. (17)	unclear	yes	yes	unclear	unclear	unclear	unclear	yes	yes	yes
Graus-Nunes F et al. (18)	yes	yes	yes	yes	unclear	unclear	unclear	yes	yes	yes
Lo CS et al. (19)	yes	yes	yes	yes	unclear	unclear	unclear	yes	yes	yes
Wang X et al. (20)	yes	yes	yes	yes	yes	yes	yes	yes	yes	yes
Zhang Y et al. (21)	yes	yes	yes	yes	unclear	unclear	unclear	yes	yes	yes
Han SX et al. (22)	yes	yes	yes	yes	unclear	unclear	unclear	yes	yes	yes
Velkoska et al. (23)	yes	yes	yes	yes	unclear	yes	yes	yes	yes	yes
Burchill L et al. (24)	yes	yes	yes	yes	unclear	yes	yes	unclear	yes	yes
Burrell LM et al. (25)	yes	yes	yes	yes	unclear	unclear	unclear	unclear	yes	yes
Ocaranza MP et al. (26)	yes	yes	yes	unclear	unclear	no	no	unclear	yes	yes
Ishiyama Y et al. (27)	yes	yes	yes	yes	unclear	yes	unclear	unclear	yes	yes
Burchill LJ et al. (28)	yes	yes	yes	unclear	unclear	unclear	unclear	no	yes	no
Burrell LM et al. (29)	yes	yes	yes	yes	unclear	yes	yes	yes	yes	yes
Yisireyili M et al. (30)	yes	yes	yes	yes	no	yes	yes	unclear	yes	yes
Ferrario CM et al. (31)	yes	yes	yes	yes	unclear	unclear	unclear	unclear	yes	yes

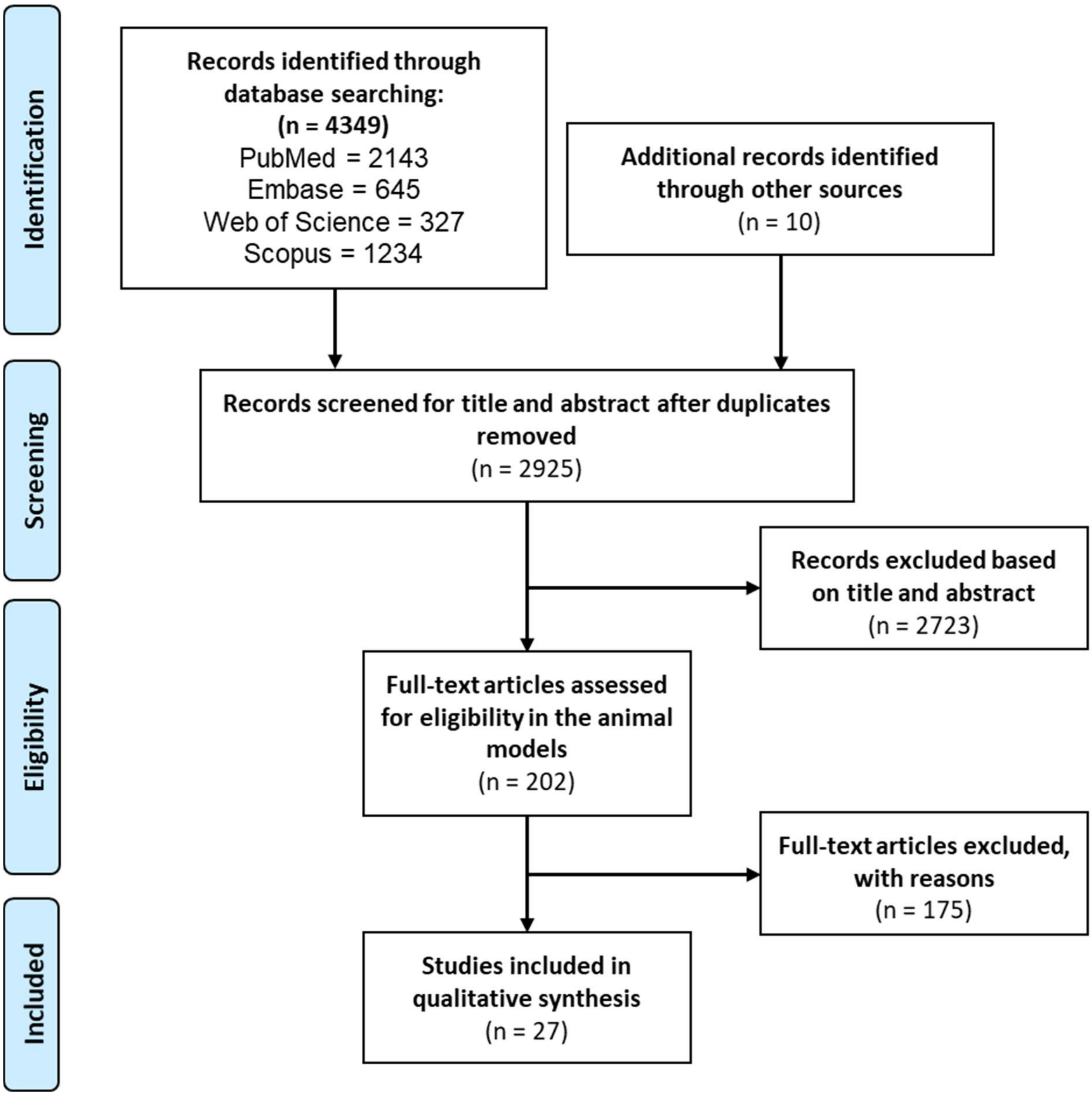

Supplementary Figure 1. PRISMA flowchart of experimental studies

References

1. Liberati A, Altman D, Tetzlaff J, Mulrow C, Gøtzsche P, Ioannidis J, et al. The PRISMA statement for reporting systematic and meta-analyses of studies that evaluate interventions: explanation and elaboration. *PLoS Medicine*. (2009) 6(7):1-28.

2. Higgins JP, Thomas J, Chandler J, Cumpston M, Li T, Page MJ, et al. *Cochrane handbook for systematic reviews of interventions*: John Wiley & Sons (2019).

3. Kriszta G, Kriszta Z, Erőss B, Pár G, Hegyi PJ, Váncsa S, et al. Effect of angiotensin-converting enzyme inhibitors and angiotensin receptor blockers on the angiotensin-converting enzyme 2 levels in experimental models, and clinical outcomes of COVID-19 in humans. (2020). doi: 10.5281/zenodo.3766469

4. Hooijmans CR, Rovers MM, De Vries RB, Leenaars M, Ritskes-Hoitinga M, Langendam MW. SYRCLE's risk of bias tool for animal studies. *BMC medical research methodology*. (2014) 14(1):43.

5. Ferrario CM, Jessup J, Chappell MC, Averill DB, Brosnihan KB, Tallant EA, et al. Effect of angiotensin-converting enzyme inhibition and angiotensin II receptor blockers on cardiac angiotensin-converting enzyme 2. *Circulation*. (2005) 111(20):2605-10. doi: 10.1161/circulationaha.104.510461

6. Cano IP, Dionisio TJ, Cestari TM, Calvo AM, Colombini-Ishikiriama BL, Faria FAC, et al. Losartan and isoproterenol promote alterations in the local renin-angiotensin system of rat salivary glands. *PloS one*. (2019) 14(5):e0217030. doi: 10.1371/journal.pone.0217030

7. Hamming I, Van Goor H, Turner A, Rushworth C, Michaud A, Corvol P, et al. Differential regulation of renal angiotensin-converting enzyme (ACE) and ACE2 during ACE inhibition and dietary sodium restriction in healthy rats. *Experimental physiology*. (2008) 93(5):631-8.

8. Jessup JA, Gallagher PE, Averill DB, Brosnihan KB, Tallant EA, Chappell MC, et al. Effect of angiotensin II blockade on a new congenic model of hypertension derived from transgenic Ren-2 rats. *American journal of physiology Heart and circulatory physiology*. (2006) 291(5):H2166-72. doi: 10.1152/ajpheart.00061.2006

9. Whaley-Connell NAC, Melvin R. Hayden, Craig S. Stump, Javad Habibi, Charles E. Wiedmeyer, Patricia E. Gallagher, E. Ann Tallant, Shawna A. Cooper, C. Daniel Link, Carlos Ferrario, and James R. Sowers. Oxidative stress and glomerular filtration barrier injury: role of the renin-angiotensin system in the Ren2 transgenic rat. *American journal of physiology Renal physiology*. (2006). doi: doi:10.1152/ajprenal.00167.2006

10. Igase M, Strawn WB, Gallagher PE, Geary RL, Ferrario CM. Angiotensin II AT1 receptors regulate ACE2 and angiotensin-(1–7) expression in the aorta of spontaneously hypertensive rats. *American Journal of Physiology-Heart and Circulatory Physiology*. (2005) 289(3):H1013-H9.

11. Agata J, Ura N, Yoshida H, Shinshi Y, Sasaki H, Hyakkoku M, et al. Olmesartan is an angiotensin II receptor blocker with an inhibitory effect on angiotensin-converting enzyme. *Hypertension research*. (2006) 29(11):865-74.

12. Takeda Y, Zhu A, Yoneda T, Usukura M, Takata H, Yamagishi M. Effects of aldosterone and angiotensin II receptor blockade on cardiac angiotensinogen and angiotensin-converting enzyme 2

expression in Dahl salt-sensitive hypertensive rats. *American journal of hypertension*. (2007) 20(10):1119-24.

13. Zhong JC, Ye JY. Telmisartan attenuates aortic hypertrophy in hypertensive rats by the modulation of ACE2 and profilin-1 expression. *Regulatory Peptides*. (2011). doi: 10.1016/j.regpep.2010.09.005

14. Yang Z, Yu X, Jiang W. Effects of enalapril on the expression of cardiac angiotensin-converting enzyme and angiotensin-converting enzyme 2 in spontaneously hypertensive rats. *Heart*. (2013) 99:A190. doi: 10.1136/heartjnl-2013-304613.529

15. Sukumaran V, Veeraveedu PT, Gurusamy N, Lakshmanan AP, Yamaguchi K, Ma M, et al. Telmisartan acts through the modulation of ACE-2/ANG 1-7/mas receptor in rats with dilated cardiomyopathy induced by experimental autoimmune myocarditis. *Life sciences*. (2012) 90(7-8):289-300. doi: 10.1016/j.lfs.2011.11.018

16. Sukumaran V, Veeraveedu PT, Lakshmanan AP, Gurusamy N, Yamaguchi Ki, Ma M, et al. Olmesartan medoxomil treatment potently improves cardiac myosin-induced dilated cardiomyopathy via the modulation of ACE-2 and ANG 1–7 mas receptor. *Free radical research*. (2012) 46(7):850-60.

17. Sukumaran V, Veeraveedu PT, Gurusamy N, Yamaguchi Ki, Lakshmanan AP, Ma M, et al. Cardioprotective effects of telmisartan against heart failure in rats induced by experimental autoimmune myocarditis through the modulation of angiotensin-converting enzyme-2/angiotensin 1-7/mas receptor axis. *International journal of biological sciences*. (2011) 7(8):1077.

18. Graus-Nunes F, Santos FD, Marinho TD, Miranda CS, Barbosa-da-Silva S, Souza-Mello V. Beneficial effects of losartan or telmisartan on the local hepatic renin-angiotensin system to counter obesity in an experimental model. *World J Hepatol*. (2019) 11(4):359-69. doi: 10.4254/wjh.v11.i4.359

19. Lo CS, Liu F, Shi Y, Maachi H, Chenier I, Godin N, et al. Dual RAS blockade normalizes angiotensin-converting enzyme-2 expression and prevents hypertension and tubular apoptosis in Akita angiotensinogen-transgenic mice. *American journal of physiology Renal physiology*. (2012) 302(7):F840-52. doi: 10.1152/ajprenal.00340.2011

20. Wang X, Ye Y, Gong H, Wu J, Yuan J, Wang S, et al. The effects of different angiotensin II type 1 receptor blockers on the regulation of the ACE-AngII-AT1 and ACE2-Ang(1-7)-Mas axes in pressure overload-induced cardiac remodeling in male mice. *Journal of molecular and cellular cardiology*. (2016) 97:180-90. doi: 10.1016/j.yjmcc.2016.05.012

21. Zhang Y, Li B, Wang B, Zhang J, Wu J, Morgan T. Alteration of cardiac ACE2/Mas expression and cardiac remodelling in rats with aortic constriction. *The Chinese journal of physiology*. (2014) 57(6):335-42. doi: 10.4077/cjp.2014.Bad268

22. Han SX, He GM, Wang T, Chen L, Ning YY, Luo F, et al. Losartan attenuates chronic cigarette smoke exposure-induced pulmonary arterial hypertension in rats: possible involvement of angiotensin-converting enzyme-2. *Toxicology and applied pharmacology*. (2010) 245(1):100-7. doi: 10.1016/j.taap.2010.02.009

23. Velkoska E, Dean RG, Burchill L, Levidiotis V, Burrell LM. Reduction in renal ACE2 expression in subtotal nephrectomy in rats is ameliorated with ACE inhibition. *Clinical Science*. (2010) 118(4):269-79.

24. Burchill L, Velkoska E, Dean RG, Lew RA, Smith AI, Levidiotis V, et al. Acute kidney injury in the rat causes cardiac remodelling and increases angiotensin-converting enzyme 2 expression. *Experimental physiology*. (2008) 93(5):622-30. doi: 10.1113/expphysiol.2007.040386

25. Burrell LM, Burchill L, Dean RG, Griggs K, Patel SK, Velkoska E. Chronic kidney disease: cardiac and renal angiotensin-converting enzyme (ACE) 2 expression in rats after subtotal nephrectomy and the effect of ACE inhibition. *Experimental physiology*. (2012) 97(4):477-85. doi: 10.1113/expphysiol.2011.063156

26. Ocaranza MP, Godoy I, Jalil JE, Varas M, Collantes P, Pinto M, et al. Enalapril attenuates downregulation of Angiotensin-converting enzyme 2 in the late phase of ventricular dysfunction in myocardial infarcted rat. *Hypertension (Dallas, Tex : 1979)*. (2006) 48(4):572-8. doi: 10.1161/01.Hyp.0000237862.94083.45

27. Ishiyama Y, Gallagher PE, Averill DB, Tallant EA, Brosnihan KB, Ferrario CM. Upregulation of angiotensin-converting enzyme 2 after myocardial infarction by blockade of angiotensin II receptors. *Hypertension (Dallas, Tex : 1979)*. (2004) 43(5):970-6. doi: 10.1161/01.HYP.0000124667.34652.1a

28. Burchill LJ, Velkoska E, Dean RG, Griggs K, Patel SK, Burrell LM. Combination renin-angiotensin system blockade and angiotensin-converting enzyme 2 in experimental myocardial infarction: implications for future therapeutic directions. *Clinical science (London, England : 1979)*. (2012) 123(11):649-58. doi: 10.1042/cs20120162

29. Burrell LM, John Risvanis, Eiji Kubota, Rachael G. Dean, Peter S. MacDonald, Sai Lu, Christos Tikellis, Sharon L. Grant, Rebecca A. Lew, A. Ian Smith, Mark E. Cooper and Colin I. Johnston. Myocardial infarction increases ACE2 expression in rat and humans. *European Heart Journal*. (2005) 26:369-75.

30. Yisireyili M, Uchida Y, Yamamoto K, Nakayama T, Cheng XW, Matsushita T, et al. Angiotensin receptor blocker irbesartan reduces stress-induced intestinal inflammation via AT1a signaling and ACE2-dependent mechanism in mice. *Brain, behavior, and immunity*. (2018) 69:167-79. doi: 10.1016/j.bbi.2017.11.010

31. Ferrario CM, VonCannon J, Ahmad S, Wright KN, Roberts DJ, Wang H, et al. Activation of the Human Angiotensin-(1-12)-Chymase Pathway in Rats With Human Angiotensinogen Gene Transcripts. *Front Cardiovasc Med*. (2019) 6:12. doi: 10.3389/fcvm.2019.00163
